# Supplementary material for: Distinct IL‐1α‐responsive enhancers promote acute and coordinated changes in chromatin topology in a hierarchical manner
Source: EMBO J. 2019 Nov 7;39(1):e101533. doi: 10.15252/embj.2019101533 (PMC6939198; doi:10.15252/embj.2019101533)

APPENDIX

Table of Contents

Appendix Figure S1. Direct modulation of heterochromatin decondensation and chromatin accessibility by NF-κB.

Appendix Figure S2. Characterization of parental and enhancer mutant HeLa compared to KB cells.

Appendix Figure S3. Cytokine array-based secretome analysis of enhancer mutant and Δ*RELA* HeLa.

Appendix Figure S4. Immuno-RNA FISH in p65 enhancer-mutant HeLa shows defects in NF-κB translocation and gene expression.

Appendix Figure S5. Impaired signal transduction in enhancer mutant HeLa.

Appendix Figure S6. Topologically-associating domain structure in proinflammatory loci across cell types.

Appendix Figure S7. *CXCL2* spatial chromatin interactions are rewired after deleting p65-binding *cis*-elements within enhancers.

Appendix Figure S8. Persistent spatial chromatin interactions in p65-knockout cells.

Appendix Table S1. Key resources.

APPENDIX FIGURE LEGENDS

Appendix Figure S1. Direct modulation of heterochromatin decondensation and chromatin accessibility by NF-κB.

(**A**) Schematic representation visualizing the U2OS cell system, where a lacO array is stably integrated into centromeric heterochromatin of chromosome 2 (Jegou, Chung et al., 2009), allowing the visualization of binding by lacI-GFP fusion proteins as a single spot (*green*).

(**B**) Representative widefield imaging of U2OS-F42B8 cells transfected with vectors encoding the indicated GFP-fusion proteins (*green*) and counterstained with Hoechst 33342 (*blue*). Scale bar: 5 µM.

(**C**) Box plots showing distributions of GFP-spot sizes from 100 cells like those in panel B. The central line marks the median; boxes indicate 25th to 75th percentiles and whiskers the 75th percentile plus 1.5 x the value of the inter-quartile distance or the 25th percentile minus 1.5 x the value of the inter-quartile distance. *: *P*-value <0.05; unpaired Student's t-test.

(**D**) Bar plots showing the fraction of cells (n=100 for each condition) transfected as in panel B and co-stained with antibodies against differentially phosphorylated RNAPII isoforms and histone marks, where the lacI-GFP fusion proteins colocalize with the immunodetected protein.

(**E**) Bar plots showing mean qPCR signal (± S.E.M.; n=3) from FAIRE experiments performed in wild-type (wt), mock transfected (empty vector), or p65-knockout HeLa cells (Δ*RELA*) in the presence or absence of TNFα (10 ng /ml, 60 min). *p< 0.05; unpaired Student's t-test.

Appendix Figure S2. Characterization of parental and enhancer mutant HeLa compared to KB cells.

(A) Representative phase contrast images of HeLa and KB cells showing their different morphology. Nuclei were stained with Hoechst 33342 (*blue*).

(B) HeLa and KB cells were left untreated or were stimulated with IL-1α for the indicated times. Expression of *IL8* and *β-actin* (*ACTB*) mRNAs were analyzed by RNA FISH. Overlay images show phase contrast (Ph) and nuclei (*blue*).

(C) Quantification of the KB RNA FISH data shown in (B). The Box plots show the distributions of FISH signals. Boundaries of the box indicate the 25th / 75th percentiles and the black lines mark medians. Whiskers (error bars) indicate the 10th / 90th percentiles and circles mark all remaining outliers. At least 250 cells were counted from each condition.

(D) 3D-DNA FISH experiments were performed for HeLa, enhancer mutant HeLa (Δp65^{eIL8}) or KB cells using labelled DNA probes that detect the *IL8* (on chr. 4) or *IL6* (on chr. 7) loci or two unrelated regions of chr. 4. Representative images are shown.

(E) The bar graphs show quantification of DNA FISH signals from 50 cells each. The percentage of cells harboring two to five copies of each chromosome is shown.

(F) Characterization of HeLa and KB cells used in this study (Kracht lab) by STR profiling reveals their identity with the original HeLa deposited at the DSMZ-German Collection of Microorganisms

and Cell Cultures; https://www.dsmz.de/dsmz). The table shows the numerical data derived from electropherograms of a range of microsatellite markers that were amplified by PCR from genomic DNA.

Appendix Figure S3. Cytokine array-based secretome analysis of enhancer mutant and Δ*RELA* HeLa cells.

(A) Design and representative example of the cytokine array used in this study. Vector controls cells were left untreated or were stimulated with IL-1α for 8 h. The supernatants were used to probe cytokine arrays coated with antibodies recognizing 80 cytokines or other inflammatory factors. After hybridizing to secondary HRP-streptavidin-conjugated antibodies signals were quantified using a chemiluminescence scanner and images were recorded. The red circles mark the top 10 most abundant factors.

(B) Quantification of inter-array normalized signals from two independent arrays. The left graph shows all signals (normalized to the mean signals of six biotinylated antibody positive controls on each array) and the right graphs shows the fold change upon IL-1α stimulation. Signals are sorted according to intensity in exp. 2. The red line marks the mean background defined by the mean of negative controls (no antibodies spotted) on all arrays. These data show that IL-6 and IL-8 are the most abundant secreted proteins and that most signals were close to or below background.

(C) IL-8 and IL-6 secretion was determined from vector controls, Δp65^{eIL8} and Δ*RELA* cell lines that were treated as described in (A). Graphs show the results from specific ELISA of supernatants (mean ± S.D., n=2). Protein concentrations of IL-8 and IL-6 were normalized to total RNA obtained from the cell pellets.

(D) All supernatants shown in (C) were also subjected to cytokine array analysis as described in detail in (A). The only additional factor that was found to be significantly present above background in the IL-1α-stimulated conditions and to be down-regulated in both independent experiments in the mutant cells was MIP3-α (CCL20). The table shows normalized signal values (secretion) and ratios of signals between the stimulated conditions.

Appendix Figure S4. Immuno-RNA FISH in p65 enhancer-mutant HeLa cells shows defects in NF-κB translocation and gene expression.

(A-C) Control (empty vector) and p65-deletion (Δp65^{eIL8}, Δp65^{eCXCL2}, and Δp65$^{eIL8+eCXCL2}$) HeLa cell lines ± IL-1α stimulation for 60 min were analyzed for nuclear translocation of p65 (*top*) and expression of *IL8* (*middle*) or *NFKBIA* (*bottom*) transcripts. Data from three independent experiments were pooled.

(A) Representative images from untreated cells and cells treated with IL-1α are shown.

(B) Subcellular localization of p65 was assessed according to four categories (*top right*) with numbers above bars indicating the total number of analyzed cells from three independent experiments. Green

labelled categories represent cells with various degrees of nuclear translocation of p65, i.e. activation of the cytosolic NF-κB pathway.

(C) The bar graphs show quantification and distribution of FISH signals. The Box plots show the distributions of FISH signals. Boundaries of the box indicate the 25th / 75th percentiles, black lines mark medians and coloured lines mark means, respectively. Whiskers (error bars) indicate the 10th / 90th percentiles and circles mark all remaining outliers.

Appendix Figure S5. Impaired signal transduction in enhancer mutant HeLa cells.

(A) Control (empty vector) or enhancer-mutant ($\Delta p65^{eIL8}$ and $\Delta p65^{eCXCL2}$) HeLa cell lines were left untreated or were stimulated with IL-1α for 30 min or 60 min. Expression of p65 NF-κB and the activation of NF-κB-IκBα, JNK and p38 MAPK was analyzed by immunoblotting of whole cell lysates using modification-site specific antibodies. Expression of factors was analyzed by antibodies recognizing the non-modified proteins. FLAG antibodies were used to determine the expression of epitope-tagged Cas9 in the stable cell lines; tubulin and β-actin levels were determined to control for equal loading. Molecular weight markers (Mr) are indicated on the left, black arrow heads mark the investigated proteins and their isoforms.

(B) Data from three independent experiments were quantified. Shown are fold changes (mean ± S.E.M.) compared to the untreated vector controls. Asterisks indicate significant changes in IL-1α-stimulated samples obtained by pairwise comparisons of either the 30 min or the 60 min time points (*$P<0.05$, unpaired, two-tailed, Student's *t*-test).

(C) Unaltered expression of p65 NF-κB upon enhancer mutations was confirmed at the *RELA* mRNA level in vector control and enhancer mutant cells before and after 60 min of IL-1α stimulation. Shown are fold changes normalized to *GUSB* compared to untreated control cells (mean ± S.E.M., n=3).

(D) Enhanced formation of inhibitory p65:IκBα complexes in $\Delta p65^{eIL8}$ mutant cells. p65 immune complexes were purified from vector control or $\Delta p65^{eIL8}$ cells before and after IL-1α treatment for 30 min or 60 min. Lysates and IPs were probed with antibodies against p65 and IκBα. At 30 min after IL-1α treatment, more IκBα was found bound to p65 confirming suppression of cytosolic NF-κB signaling. Molecular weight markers (Mr) are indicated on the left, black arrow heads mark the investigated proteins and their isoforms.

Appendix Figure S6. Topologically-associating domain structure in proinflammatory loci across cell types.

(**A**) High-resolution Hi-C (from http://promoter.bx.psu.edu/hi-c/) depicting the TAD encompassing the *IL8* and *CXCL1-6* IL-1α-inducible loci on human chromosome 4 in 7 ENCODE cell lines.

(**B**) As in panel A, but for TADs in the extended *BMP4*/*SAMD4A* loci on chromosome 14.

Appendix Figure S7. *CXCL2* spatial chromatin interactions are rewired after deleting p65-binding *cis*-elements within enhancers.

(**A**) i4C profiles in the 1 Mbp around the *CXCL2* locus on chromosome 4 (*ideogram*) from control (empty vector) and enhancer-mutant ($\Delta p65^{eIL8}$ and $\Delta p65^{eCXCL2}$) HeLa lines ± IL-1α stimulation for 60 min. The average of two biological replicates is plotted, generated using the *CXCL2* promoter (*blue highlight*) or enhancer (*pink highlight*) as a viewpoint. Below each profile significant strong (*brown*), medium (*red*) or weaker interactions (*orange*) called via *foursig* are indicated. All profiles are shown aligned to gene models (*blue*) and to CTCF, H3K4me1, H3K4me3, H3K27ac, and RNA polymerase II ENCODE ChIP-seq profiles from HeLa-S3 cells. The breadth of topologically-associating domains (TADs) in the locus is indicated above (*rectangles*).

(**B**) Meta-plots showing coverage of H3K27ac ChIP-seq signal at i4C fragments ± 1 kbp contacted by the *CXCL2* promoter or enhancer in control cells (empty vector) in the presence (*magenta*) or absence (*gray*) of IL-1α stimulation for 60 min, and in enhancer-mutant cells ($\Delta p65^{eIL8}$, *blue*; $\Delta p65^{eCXCL2}$, *green*) after IL-1α stimulation.

Appendix Figure S8. Persistent spatial chromatin interactions in p65-knockout cells.

(**A**) i4C profiles in the 1 Mbp around the *CXCL2* and *IL8* loci on chromosome 4 (*ideogram*) from p65-knockout (ΔRELA) HeLa cells ± IL-1α stimulation for 60 min. Data were generated using the *CXCL2* enhancer (*blue highlight*) or *IL8* promoter (*pink highlight*) as viewpoints, and profiles are shown aligned to gene models (*blue*) and to CTCF, H3K4me1, H3K4me3, H3K27ac, and RNA polymerase II ENCODE ChIP-seq profiles from HeLa-S3 cells. The breadth of topologically-associating domains (TADs) in the locus is indicated above (*rectangles*).

(**B**) As in panel A, but using the promoters of the inducible *BMP4* (*pink highlight*) and *SAMD4A* genes (*blue highlight*) on chromosome 14 as viewpoints ± TNFα stimulation for 60 min.

Appendix Table S1. Key resources.

REAGENT or RESOURCE	SOURCE	IDENTIFIER
Antibodies		
β-Actin (mouse)	Santa Cruz Biotechnology	Cat#sc-4778
CRISPR-Cas9 (mouse)	Abcam	Cat#ab191468
CTCF (rabbit)	Millipore	Cat#07-729
Dylight 488 donkey anti mouse IgG	ImmunoReagents	Cat#DkxMu-003D488NHSX
FLAG (mouse)	Sigma-Aldrich	Cat#F1804
goat-anti-rabbit IgG, Cy3	Diagenode	Cat#111-165-003
H3 (rabbit)	Abcam	Cat#ab1791
H3K27ac (rabbit)	Diagenode	Cat#Pab-174-050
H3K27me3 (rabbit)	Millipore	Cat#07-449
H3K36ac (rabbit)	Diagenode	Cat#C15410307
H3K4me1 (rabbit)	Abcam	Cat#ab8895
HRP-coupled anti rabbit IgG	Thermo Fisher Scientific	Cat#31460
HRP-coupled anti-mouse IgG	Dako	Cat#P0447
HRP-coupled anti-rabbit IgG	Dako	Cat#P0448
IκBα (rabbit)	Cell Signaling	Cat#9242
JNK (rabbit)	Santa Cruz Biotechnology	Cat#sc-571
NFκB p65 (F-6) (mouse)	Santa Cruz Biotechnology	Cat#sc-8008
NFκB p65 (rabbit)	Santa Cruz Biotechnology	Cat#sc-372
Normal rabbit IgG	Santa Cruz Biotechnology<br>Cell Signaling	Cat#sc-2027<br>Cat#2729
p38 (rabbit)	Cell Signaling	Cat#9212
phospho-IκBα S32 (rabbit)	Cell Signaling	Cat#2859
phospho-JNK T183/Y185 (rabbit)	Cell Signaling	Cat#9251
phospho-NFκB p65 S536 (rabbit)	Cell Signaling	Cat#3033
phospho-p38 T180/Y182 (rabbit)	Zymed	Cat#36-8500
phospho-Pol II S2 (rabbit)	Abcam	Cat#ab5095
phospho-Pol II S5 (rabbit)	Abcam	Cat#ab5131
Puromycin 3RH11 (mouse)	Kerafast	Cat#EQ0001
RNA-Pol II (mouse)	Millipore	Cat#17-620
TrueBlot HRP conjugated anti rabbit IgG	Rockland	Cat#18-8816-31
Tubulin (mouse)	Santa Cruz Biotechnology	Cat#sc-8035
Chemicals, Reagents and Recombinant Proteins		
Paraformaldehyde solution 4%	Santa Cruz Biotechnology	Cat#sc-281692
Hoechst 33342	Thermo Fisher Scientific	Cat#H3570
Human recombinant IL-1α	Expressed in and purified from *E.Coli*	(Rzeczkowski, Beuerlein et al., 2011)
Human recombinant TNFα	ImmunoTools	Cat#11343018
Lithium Chloride Precipitation Solution	Thermo Fisher Scientific	Cat#AM9480
Normal Donkey Serum	Jackson ImmunoResearch	Cat#017-000-121
Puromycin	InvivoGen	Cat#ant-pr-1
Roti-Load	Carl Roth	Cat#K929.1
Saponin	Sigma-Aldrich	Cat#S4521-10G
TrueBlot anti rabbit Ig IP Beads	Rockland	Cat#00-8800-25
FCS/FBS	PAN Biotech	Cat#1502-P110704<br>Cat#P40-47500<br>P160204
Critical Commercial Assays		
Fast SYBR Green PCR Master Mix	Applied Biosystems	Cat#4385612
DuoSet® ELISA IL-6	R&D Systems	Cat#DY206
DuoSet® ELISA IL-8	R&D Systems	Cat#DY208

MinElute PCR Purification Kit	Qiagen	Cat# 28004
NEBNext® High-Fidelity 2X PCR Master Mix	NEB	Cat#M0541S
Nextera DNA Library Preparation Kit	Illumina	Cat# FC-121-1030
NucleoBond PC500	Macherey&Nagel	Cat#740574.50
NucleoSpin Gel and PCR Clean-Up	Macherey&Nagel	Cat#740609.250
NucleoSpin RNA II	Macherey&Nagel	Cat#740955.250
NucleoSpin Tissue	Macherey&Nagel	Cat#740952.50)
Quantigene View RNA FISH Cell Assay Kit	Affymetrix	Cat#QVC0001
RayBio® C-Series Human Cytokine Antibody Array C5	RayBiotech	Cat#AAH-CYT-5-8
Venor®GeM Classic	minerva biolabs	Cat#11-1100 MB
TaqMan Fast Universal PCR Master Mix	Applied Biosystems	Cat#4352042
Enzymes		
DNaseI	Thermo Fisher Scientific	Cat#EN0521
FastDigest BbsI	Thermo Fisher Scientific	Cat#FD1014
GoTaq G2 Flexi DNA Polymerase	Promega	Cat#M7805
M-MuLV Reverse Transcriptase	Thermo Fisher Scientific	Cat#EP0352
RevertAid Reverse Transcriptase,	Thermo Fisher Scientific	#EP0441
Plasmid-Safe ATP-Dependent DNase	Biozym	#161010
Proteinase K (600-1000 U/ml)	Fermentas	#EO0491
RNase A (10 mg/ml)	Fermentas	#EN0531
T4 DNA Ligase	Thermo Fisher Scientific	Cat#EL0014
T4 Polynucleotid Kinase	Thermo Fisher Scientific	Cat#EK0031
Inhibitors		
Actinomycin D	Sigma-Aldrich	Cat#A1410
Leupeptin Hemisulfat	Carl Roth	Cat#CN33.2
Microcystin	Enzo Life Sciences	Cat#ALX-350-012-M001
Pepstatin A	Applichem	Cat#A2205
PMSF	Sigma-Aldrich	Cat#P-7626
Protease inhibitor cocktail tablets	Roche	Cat#11873580001
RiboLock RNase Inhibitor	Thermo Fisher Scientific	Cat#E00381
5Z-7-oxozeaenol	Tocris Bioscience	Cat#66018-38-0
Plasmids		
pSpCas9(BB)-2A-Puro (pX459) V2.0	Addgene	Cat#62988
pSpCas9(BB)-2A-Puro (pX459)	Addgene	Cat#48139
pSpCas9(BB)-2A-GFP (pX458)	Addgene	Cat#48138
pX459-sg1Δp65	Cloned by AG Schmitz	
pX459-sg1IL8Promoter	Cloned by AG Kracht	
pX459 V2.0-sg2IL8Promoter	Cloned by AG Kracht	
pX459-sg1IL8Enhancer	Cloned by AG Kracht	
pX459-sg2IL8Enhancer	Cloned by AG Kracht	
pX459-sg3IL8Enhancer	Cloned by AG Kracht	
pX59-sg1CXCL2Enhancer	Cloned by AG Kracht	
pX459-sg2CXCL2Enhancer	Cloned by AG Kracht	
lenti sgRNA(MS2)-zeo backbone	Addgene	Cat#61427

lenti sgRNA(MS2)-zeo-sg1IL8Promoter	Cloned by AG Kracht	
lenti sgRNA(MS2)-zeo-sg3IL8Promoter	Cloned by AG Kracht	
lenti sgRNA(MS2)-zeo-sg4IL8Promoter	Cloned by AG Kracht	
lenti sgRNA(MS2)-zeo-sg1IL8Enhancer	Cloned by AG Kracht	
lenti sgRNA(MS2)-zeo-sg2IL8Enhancer	Cloned by AG Kracht	
lenti sgRNA(MS2)-zeo-sg3IL8Enhancer	Cloned by AG Kracht	
lenti sgRNA(MS2)-zeo-sg4IL8Enhancer	Cloned by AG Kracht	
lenti sgRNA(MS2)-zeo-sg5IL8Enhancer	Cloned by AG Kracht	
lenti sgRNA(MS2)-zeo-sg6IL8Enhancer	Cloned by AG Kracht	
lenti sgRNA(MS2)-zeo-sg7IL8Enhancer	Cloned by AG Kracht	
lenti sgRNA(MS2)-zeo-sg8IL8Enhancer	Cloned by AG Kracht	
lenti sgRNA(MS2)-zeo-sg1CXCL2Promoter	Cloned by AG Kracht	
lenti sgRNA(MS2)-zeo-sg2CXCL2Promoter	Cloned by AG Kracht	
lenti sgRNA(MS2)-zeo-sg3CXCL2Promoter	Cloned by AG Kracht	
lenti sgRNA(MS2)-zeo-sg4CXCL2Promoter	Cloned by AG Kracht	
lenti sgRNA(MS2)-zeo-sg5CXCL2Promoter	Cloned by AG Kracht	
lenti sgRNA(MS2)-zeo-sg1CXCL2Enhancer	Cloned by AG Kracht	
lenti sgRNA(MS2)-zeo-sg2CXCL2Enhancer	Cloned by AG Kracht	
lenti sgRNA(MS2)-zeo-sg3CXCL2Enhancer	Cloned by AG Kracht	
lenti dCAS9-VP64_Blast	Addgene	Cat#61425
lenti MS2-P65-HSF1_Hygro	Addgene	Cat#61426
pcDNA3-LacI-eGFP	MCS (empty vector), GFP-tag modified by L.Schmitz	PMID: 19211845,
pcDNA3-LacI-eGFP	MDB2a, GFP-tag	PMID: 19211845
pcDNA3-LacI-eGFP	VP16, GFP	PMID: 19211845
pcDNA3-LacI-eGFP	p65 WT, GFP-tag	T.Riedlinger
Oligonucleotides (5‘-3‘-sequence, restriction site overhangs in red)		
sgRNA OLIGOS	Forward	Reverse
sg1 p65 knockout chr11:65429474-65429496	CACCGGCTTCCGCTACAAGTGCGA	AAACTCGCACTTGTAGCGGAAGCC
sg1 IL8 promoter chr4:74606177-74606199	CACCGATTCCACGATTTGCAACTGA	AAACTCAGTTGCAAATCGTGGAATC
sg2 IL8 promoter chr4:74606225-74606247	CACCGGGGTGCATAAGTTCTCTAGT	AAACACTAGAGAACTTATGCACCCC
sg3 IL8 promoter chr4:74606204-74606226	CACCGTCTGACATAATGAAAAGATG	AAACCATCTTTTCATTATGTCAGAC
sg4 IL8 promoter chr4:74606147-74606169	CACCGGATGACTCAGGTTTGCCCTG	AAACCAGGGCAAACCTGAGTCATCC
sg1 IL8 enhancer chr4:74591837-74591859	CACCGACCATGTGTGGAATTTCCCA	AAACTGGGAAATTCCACACATGGTC
sg2 IL8 enhancer	CACCGATAAGGAAAGGGG	AAACTTTGAATCC

chr4:74591800-74591822	ATTCAAA	CCTTTCCTTATC
sg3 IL8 enhancer chr4:74591769-74591791	CACCGTATTCTTGAGGCATCTGTGA	AAACTCACAGATGCCTCAAGAATAC
sg4 IL8 enhancer chr4:74591892-74591914	CACCGGTCAGCCAAAGACATTGCAC	AAACGTGCAATGTCTTTGGCTGACC
sg5 IL8 enhancer chr4:74591691-74591713	CACCGGGCTCCTAATGGCAAAGTTA	AAACTAACTTTGCCATTAGGAGCCC
sg6 IL8 enhancer chr4:74591501-74591523	CACCGGACCTAATAGAAGCTCTTTC	AAACGAAAGAGCTTCTATTAGGTCC
sg7 IL8 enhancer chr4:74591326-74591348	CACCGAACGAAACAAGAGCACTTTA	AAACTAAAGTGCTCTTGTTTCGTTC
sg8 IL8 enhancer chr4:74591917-74591939	CACCGTTTATACTGAGTCACCTCTC	AAACGAGAGGTGACTCAGTATAAAC
sg1 CXCL2 promoter chr4:74965059-74965081	CACCGGGGCACTCACGAGTGACGTC	AAACGACGTCACTCGTGAGTGCCCC
sg2 CXCL2 promoter chr4:74965007-74965029	CACCGATCGCCTTCCTTCCGAACTC	AAACGAGTTCGGAAGGAAGGCGATC
sg3 CXCL2 promoter chr4:74964960-74964982	CACCGAAGCCCGGAGTCCCGGGCCA	AAACTGGCCCGGGACTCCGGGCTTC
sg4 CXCL2 promoter chr4:74964919-74964941	CACCGTAAAAGGGGTTCGCCGTTCT	AAACAGAACGGCGAACCCCTTTTAC
sg5 CXCL2 promoter chr4:74964840-74964862	CACCGGGCTCAGCAGGCGGTTCGAG	AAACCTCGAACCGCCTGCTGAGCCC
sg1 CXCL2 enhancer chr4:74982878-74982900	CACCGGACAATGTCATTTGTTGTCA	AAACTGACAACAAATGACATTGTCC
sg2 CXCL2 enhancer chr4:74982807-74982829	CACCGTTGGAGAAAGGGATTTTAAA	AAACTTTAAAATCCCTTTCTCCAAC
sg3 CXCL2 enhancer chr4:74982854-74982876	CACCGTATGAAGCCCCGTCCACTTG	AAACCAAGTGGACGGGGCTTCATAC
sg SAMD4A enhancer (eSAM1) chr14: 54,627,167-54,628,309	AAACTCAGTACTTACCCTACCCTAC	AAACGACGGTCTAGGTGGTGCTGC
sg BMP4 enhancer (eBMP) chr14: 53,890,163-53,891,635	AAACATGTAGTCTGAATCCAGAGC	AAACCCAGTATATTAAGGGCAGTTC
OLIGOS FOR g/cDNA PCR	Forward	Reverse
PCR gDNA flanking IL8 enhancer deletion chr4:74591354+74592122	GCACATTGCTCCCTGACTTC	GGACGGTGACCTCTGTGTAA
PCR gDNA flanking CXCL2 enhancer deletion chr4:74982502-74983250	GCCCTACTTGAACGCTTCCT	TTCCTTGGCTTTGGTTCAAAGAG
PCR gDNA flanking IL8 promoter deletion chr4:74605767+74606731	ATGCCCCCTAAGAGCAGTAA	ACCGTGGTTCTCAATAGGACA
PCR gDNA SAMD4A enhancer deletion chr14: 54,627,167-54,628,309	TGTTCAACACATCTGGATTCTGG	GCTTGACTTTCAGAGCATCAGC
PCR gDNA BMP4 enhancer deletion chr14: 53,890,163-53,891,635	GTGAGGAAAGTGGTGGGAAA	CTGTGGGCTGTGTACCTGATT
PCR cDNA dCas9	CGAGATCAACAACTACCACC	AAGAAGTACTTGGCGGTAGC
OLIGOS FOR Sanger Sequencing	Forward	
pX459 sgRNA integration	GCATATACGATACAAGGCTG	
OLIGOS FOR ChIP-qPCR		
ChIP IL8 promoter chr4:74606096+74606266	AAGAAAACTTTCGTCATACTCCG	TGGCTTTTTATATCATCACCCTAC
ChIP IL8 enhancer chr4:74591806+74592316	AAAGGGGATTCAAAGGGAGA	GGTTGCTGAAACACAGCTCA
ChIP CXCL2 promoter	GTCAGACCCGGACGTCACT	ACCCCTTTTATGC

chr4:74964932-74965088		ATGGTTG
ChIP CXCL2 enhancer chr4:74982786+74982953	AGGCCTCTGTGATGGAAAT G	TCCTTCTGTTGGCT CCATTC
ChIP IL6 promoter chr7:22766625+22766816	TCCCCCTAGTTGTGTCTTG C	GCCTCAGACATCT CCAGTCC
ChIP CCL20 promoter chr2:228678390+228678549	CTTCGCACCTTCCCAATA	CCTGGGATGGCCC TATTTAT
ChIP NFKBIA promoter chr14:35873991-35874141	CTCATCGCAGGGAGTTTCT C	CTGGCTGGGGATT TCTCTG
ChIP CXCL1 promoter chr4:74734934+74735098	ATCAGTGGACCCCCACAC	ATCCGCGAACCCC TTTTAT
ChIP CXCL3 promoter chr4:74904417-74904586	GGCGTAGGCGTCACCAG	GATCGGCGAACCC TTTTTAT
OLIGOS FOR ATAQ-Seq		
ATAC-Seq Custom Nextera Primer 1 (I5)	AATGATACGGCGACCACC GAGATCTACACTCGTCGGC AGCGTCAGATGTG	
ATAC-Seq Custom Nextera Primer 2 (I7, different primer for each sample):		
N701 (KB untreated)	CAAGCAGAAGACGGCATA CGAGATTCGCCTTAGTCTC GTGGGCTCGGAGATGT	
N702 (KB IL-1)	CAAGCAGAAGACGGCATA CGAGATCTAGTACGGTCTC GTGGGCTCGGAGATGT	
N704 (KB 5Z-7-Oxozeanol)	CAAGCAGAAGACGGCATA CGAGATGCTCAGGAGTCTC GTGGGCTCGGAGATGT	
N705 (KB combination)	CAAGCAGAAGACGGCATA CGAGATAGGAGTCCGTCTC GTGGGCTCGGAGATGT	
OLIGOS FOR FAIRE-qPCR	Forward	Reverse
hChIP neg. ctr. chr12:6637421+6637594	ATGGTTGCCACTGGGGATC T	TGCCAAAGCCTAG GGGAAGA
ACTB-ChIP-TSS chr7:5570290+5570428	AAAGGCAACTTTCGGAAC GG	TTCCTCAATCTCGC TCTCGC
hIL8 ChIP chr4:74606145+74606283	GTGATGACTCAGGTTTGCC CT	CTTATGGAGTGCT CCGGTGG
hNFκB1 ChIP chr4:103422452+103422558	TCTCTCGACGTCAGTGGGA ATTT	TGGCGAAACCTCC TCTTCCTG
hICAM1 ChIP chr19:10381498+10381655	CGCGGTGTAGACCGTGATT	GAGAGGGTCATCC TCCCTCG
hBBC3 ChIP chr19:47730990-47731065	CGGGAGGACTGTCAACTCT G	CCTGGCCAAAGAG ATGCAAAT
CCL20TSS chr2:228678390+228678549	CTTCGCACCTTCCCAATAT G	CCTGGGATGGCCC TATTTAT
OLIGOS FOR i4C EXPERIMENTS (w/o Illumina adapters)	Forward	Reverse
i4C-pIL8	TAGAGCCTGACCAGCCTGC AATGGCTGCAGGAATTC	
i4C-eIL8	GCTAGCGGCCAAGTGGGG AGAGAATTT	
i4C-pCXCL2	AGCTAGGGAGAGATCATG GCAGTAAGGCAGGAATTT	
i4C-eCXCL2	AGCTAGGTCGGCTAGCCAA AAAATGTCACCTAAATTT	

SAMD4A-e1_WT	TCTAGAGGCTTTTATTTTTG TCAATGGTTGG	GATGTGTGCTAGA TATTCAGGAGCT
SAMD4A-e1_DEL	TTAGAGCCACCACCTAGAC CTGAAGGA	GAAGTGTGGCTCC TTAATCCAG
BMP4e_WT	TTAGGACTGGGGGTAGTAG ATAAAGGATTAGA	GATCACACCAGTA ATCCCAGCAC
BMP4e_DEL	TGTAAGCGCCCTTAATATA CCATGGGATGAAAT	GATCACACCAGTA ATCCCAGCAC
MCi4C-pSAMD4A	CGCAGCCGAACTTTCTTTG	GACGGGTCCGGGT GAATTT
MCi4C-eSAMD4A	TAAATTGGGAGGCCAGTCA C	AGAGCCCGATGGA AAAACAC
MCi4C-eBMP4	CAAAGCTACAGGGACTTTG GAG	TGGATTCAGACTA CATGGGATG
TaqMan Gene Expression Assays		
ACTB	Thermo Fisher Scientific	Cat#Hs99999903_m1
GUSB	Thermo Fisher Scientific	Cat#Hs99999908_m1
IL6	Thermo Fisher Scientific	Cat#Hs00174131_m1
IL8	Thermo Fisher Scientific	Cat#Hs00174103_m1
NFKBIA	Thermo Fisher Scientific	Cat#Hs00153283_m1
CXCL1	Thermo Fisher Scientific	Cat#Hs00236937_m1
CXCl2	Thermo Fisher Scientific	Cat#Hs00236966_m1
CXCL3	Thermo Fisher Scientific	Cat#Hs00171061_m1
CCL20	Thermo Fisher Scientific	Cat#Hs00171125_m1
RELA (p65)	Thermo Fisher Scientific	Cat#Hs00153294_m1
Probe sets for RNA *in situ* hybridisation		
Human *IL8* Type 4	Affymetrix	Cat#VA4-13193 or VA1-13103
Human *NFKBIA* Type 6	Affymetrix	Cat#VA6-17971
Human *ACTB* Type 4	Affymetrix	Cat#VA4-10293
Human *CXCL8*-intronic Type 1	Affymetrix	VA1-6000437
Human *CXCL2*-intronic Type 6	Affymetrix	VA6-6000438
Software		
Leica FW4000 v. 1.2.1		
Leica LasX v. 1.5.1.13187		
Adobe Photoshop v. 7.0		
Bio-Rad Image Lab v. 5.2.1 build 11		
Duolink ImageTool v. 1.0.1.2		
SigmaPlot 11.2.05		
MS EXCEL 2013		
GraphPadPrism 6.07		
Cell lines		
name	Lab name	Transfected with
HUVEC	pooled HUVEC	parental
HeLa	HeLa HG	parental
KB	KB	parental
hTERT- RPE-1	RPE-1	parental
HeLa empty vector (pX459, vs.1.0)	U.Tenekeci, Kracht lab	pX459 vs.1.0
HeLa empty vector (pX459, vs. 2.0), Fig. EV4	ST110, S.Weiterer, Kracht lab	pX459 vs.2.0
HeLa $\Delta p65^{eIL8}$	Mix12	sg1 IL8 enhancer + sg2 IL8 enhancer + sg3 IL8 enhancer

HeLa Δp65^{eCXCL2}	Mix66	sg1 CXCL2 enhancer + sg2 CXCL2 enhancer
HeLa Δp65$^{eIL8+eCXCL2}$	Mix73	sg1 IL8 enhancer + sg2 IL8 enhancer + sg3 IL8 enhancer + sg1 CXCL2 enhancer + sg2 CXCL2 enhancer
HeLa Δp65^{pIL8}	Mix110	sg1 IL8 promoter + sg2 IL8 promoter
HeLa ΔRELA	M. Poppe, Kracht lab (used for IL-1 experiments	sg1 p65 knockout (Δp65)
HeLa ΔRELA	T. Riedlinger, Schmitz lab (used for TNFα experiments	sg1 p65 knockout (Δp65)
U2OS	T. Riedlinger, Schmitz lab	

REFERENCES

Jegou T, Chung I, Heuvelman G, Wachsmuth M, Gorisch SM, Greulich-Bode KM, Boukamp P, Lichter P, Rippe K (2009) Dynamics of telomeres and promyelocytic leukemia nuclear bodies in a telomerase-negative human cell line. Mol Biol Cell 20: 2070-82

Rzeczkowski K, Beuerlein K, Muller H, Dittrich-Breiholz O, Schneider H, Kettner-Buhrow D, Holtmann H, Kracht M (2011) c-Jun N-terminal kinase phosphorylates DCP1a to control formation of P bodies. J Cell Biol 194: 581-96

Appendix Fig S1

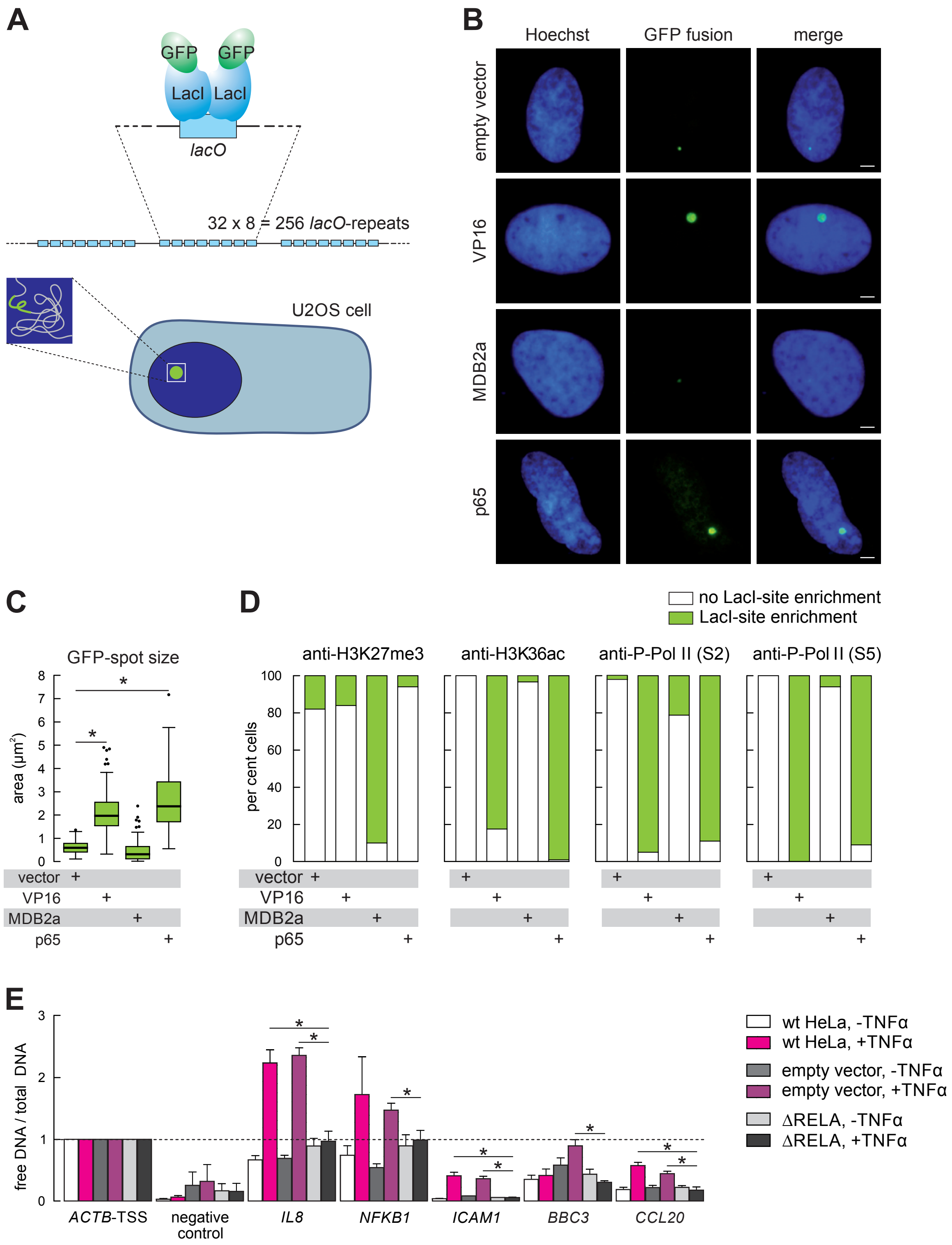

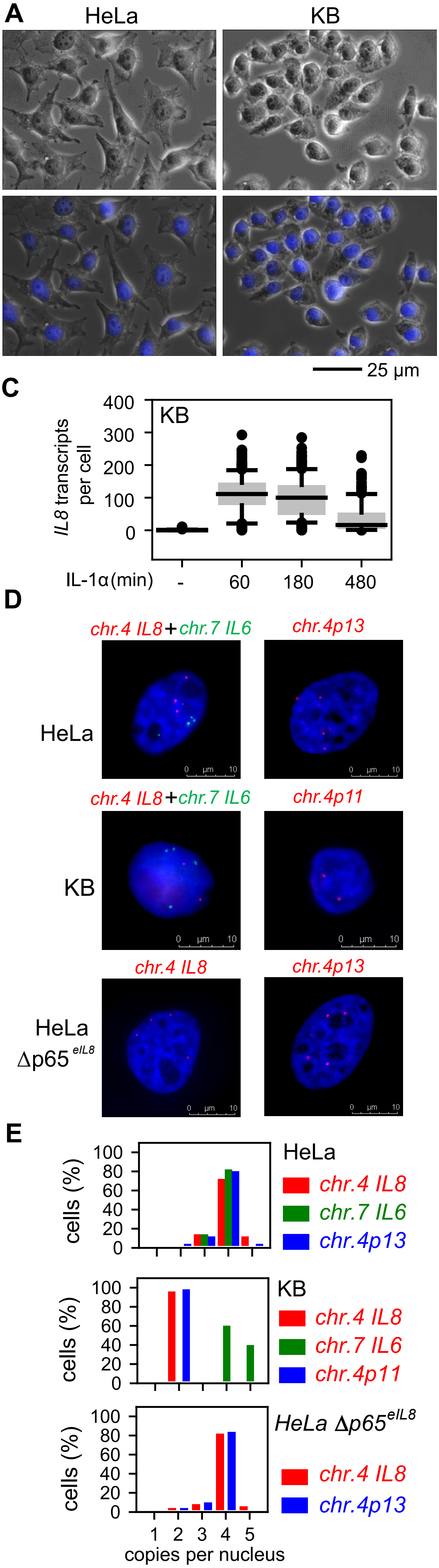

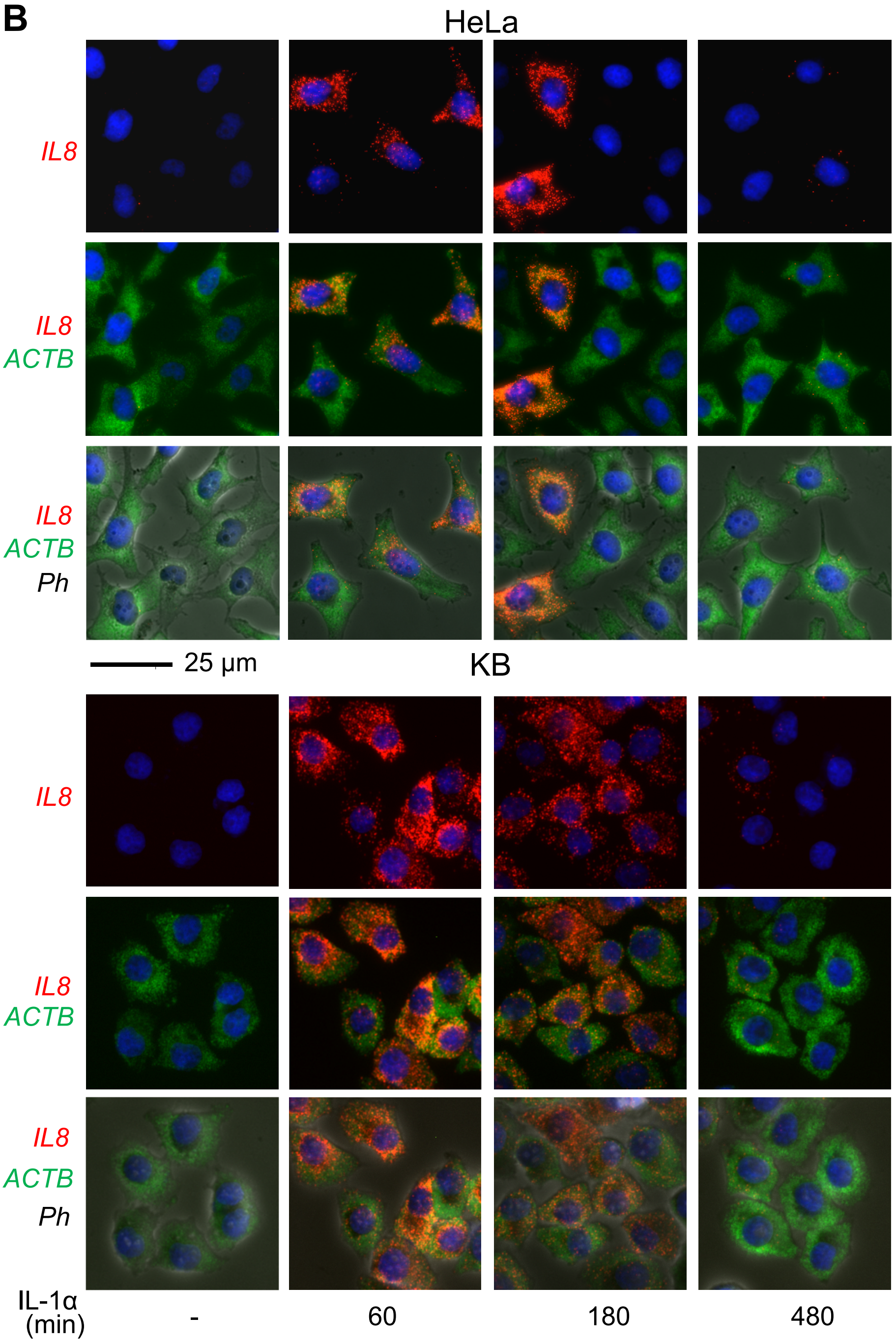

F

short tandem repeat (STR) microsatellite profiles of HeLa and KB cells

	Cell line	Date	D5	D5'	D13	D13'	D7	D7'	D16	D16'	vWA	vWA'	TH01	TH01'	TPOX	TPOX'	CSF1	CSF1'	Amel	Amel'	Date Animal-PCR:	M	R	CH	SH
DSMZ (ACC 57)	HELA	23_01_2018	11	12	12	13.3	8	12	9	10	16	18	7	7	8	12	9	10	X	X	23_01_2018	-	-	-	-
DSMZ (ACC 136)	KB	17_07_2008	11	12	12	13.3	8	12	9	10	16	18	7	7	8	12	9	10	X	X	17_07_2008	-	-	-	-
Kracht lab	HeLa	22_01_2019	11	12	13	13.3	8	12	9	10	16	18	7	7	12	12	9	10	X	X	22_01_2019	-	-	-	-
Kracht lab	KB cells	20_05_2019	11	12	12	13.3	8	12	9	10	16	18	7	7	8	12	9	10	X	X	20_05_2019	-	-	-	-

Appendix Fig S2

Appendix Fig S3

A

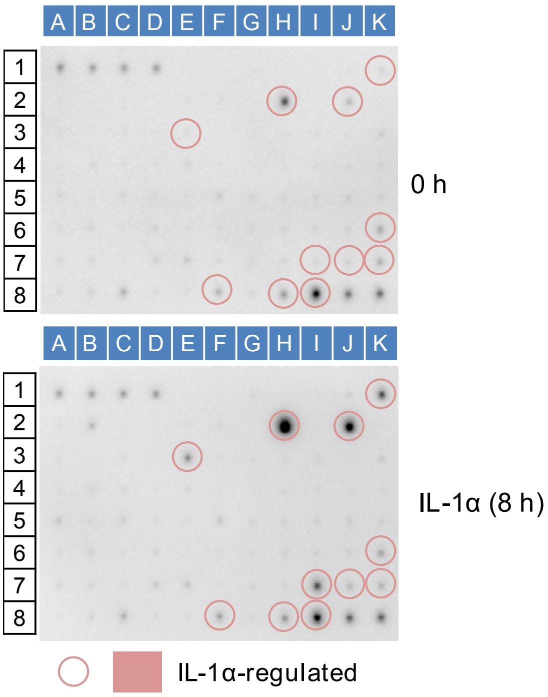

	A	B	C	D	E	F	G	H	I	J	K
1	POS	POS	POS	POS	NEG	NEG	ENA-78 (CXCL5)	G-CSF	GM-CSF	GROα/β/γ	GROα (CXCL1)
2	I-309 (CCL1)	IL-1α (IL-1 F1)	IL-1β (IL-1 F2)	IL-2	IL-3	IL-4	IL-5	IL-6	IL-7	IL-8 (CXCL8)	IL-10
3	IL-12 p40/p70	IL-13	IL-15	IFN-γ	MCP-1 (CCL2)	MCP-2 (CCL8)	MCP-3 (CCL7)	M-CSF	MDC (CCL22)	MIG (CXCL9)	MIP-1β (CCL4)
4	MIP-1δ	RANTES (CCL5)	SCF	SDF-1 α	TARC (CCL17)	TGFβ1	TNFα	TNFβ (TNFSF1B)	EGF	IGF-1	Angiogenin
5	OSM	TPO	VEGF-A	PDGF-BB	Leptin	BDNF	BLC (CXCL13)	Ckβ 8-1 (CCL23)	Eotaxin-1 (CCL11)	Eotaxin-1 (CCL24)	Eotaxin-3 (CCL26)
6	FGF-4	FGF-6	FGF-7 (KGF)	FGF-9	FLT-3 Ligand	Fractalkine (CX3CL1)	GCP-1 (CXCL6)	GDNF	HGF	IGFBP-1	IGFBP-2
7	IGFBP-3	IGFBP-4	IL-16	IP-10 (CXCL10)	LIF	LIGHT (TNFSF14)	MCP-4 (CCL13)	MIF	MIP-3α	NAP-2 (CXCL7)	NT-3
8	NT-4	OPN (SPP1)	OPG (TNFRSF11)	PARC	PLGF	TGFβ2	TGFβ3	TIMP-1	TIMP-2	POS	POS

B

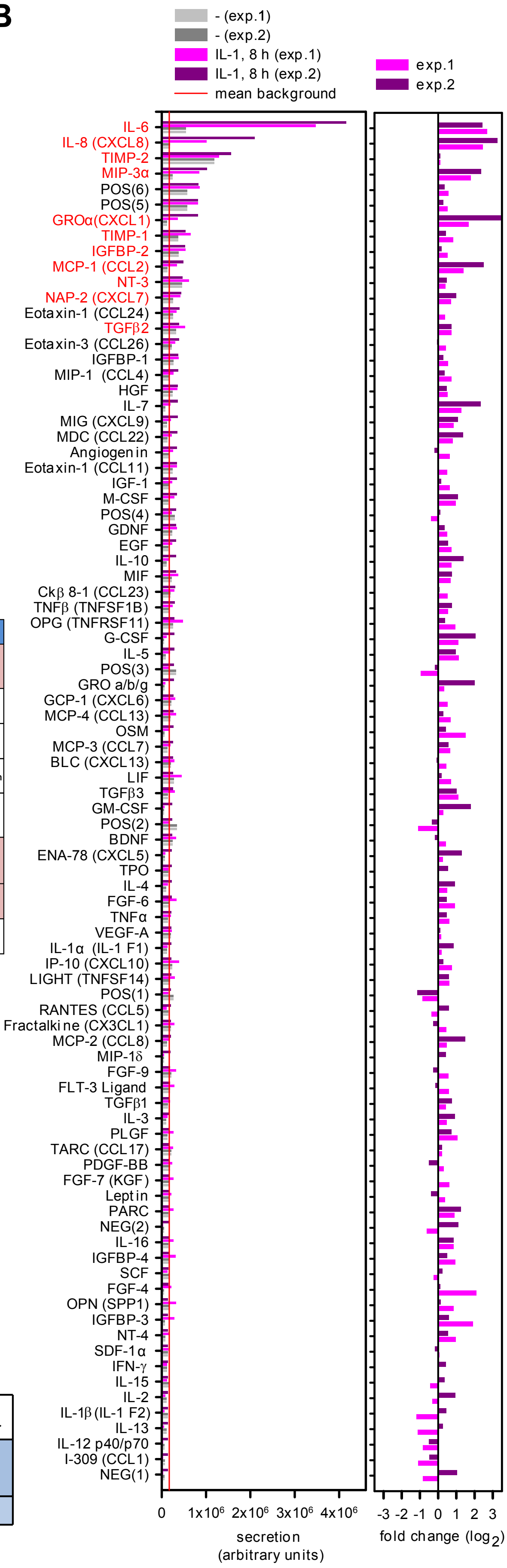

C

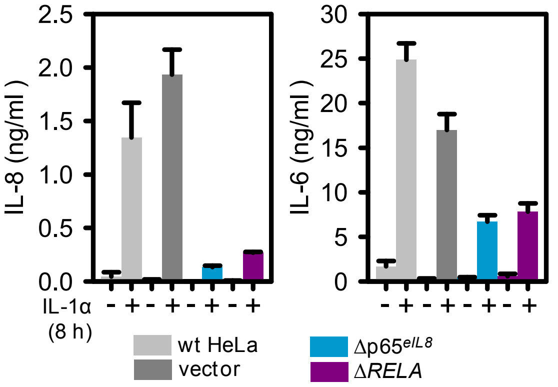

D

		secretion (arbitrary units x 10^{-3})			Δp65^{eIL8} / vector	*ΔRELA* / vector
MIP-3α (CCL-20)	exp.1	1579.9	805.1	526.5	-1.0	-1.6
	exp.2	1405.4	388.8	592.7	-1.9	-1.2

vector + IL-1α; Δp65^{eIL8} + IL-1α; *ΔRELA* + IL-1α

Appendix Fig S4

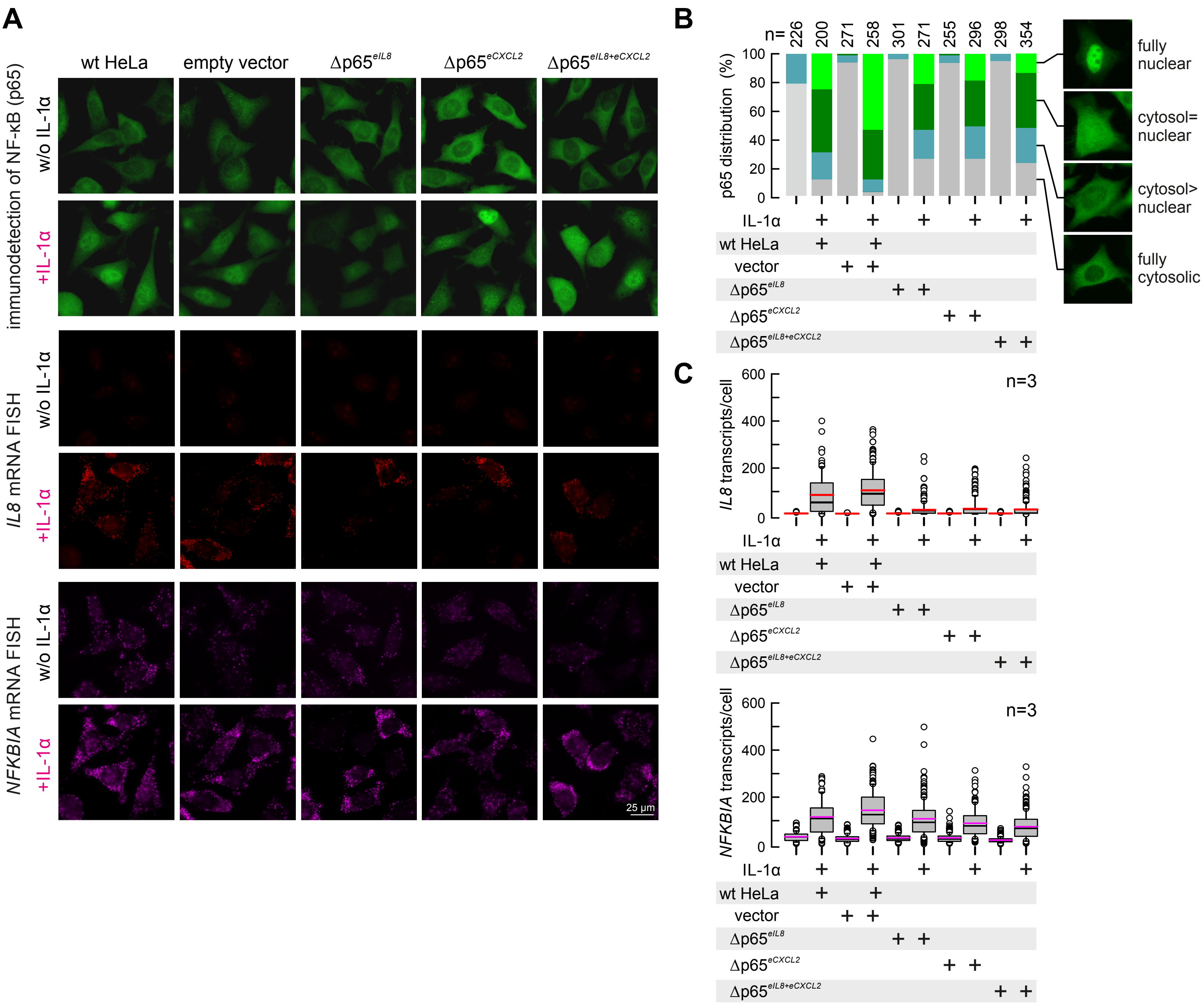

Appendix Fig S5

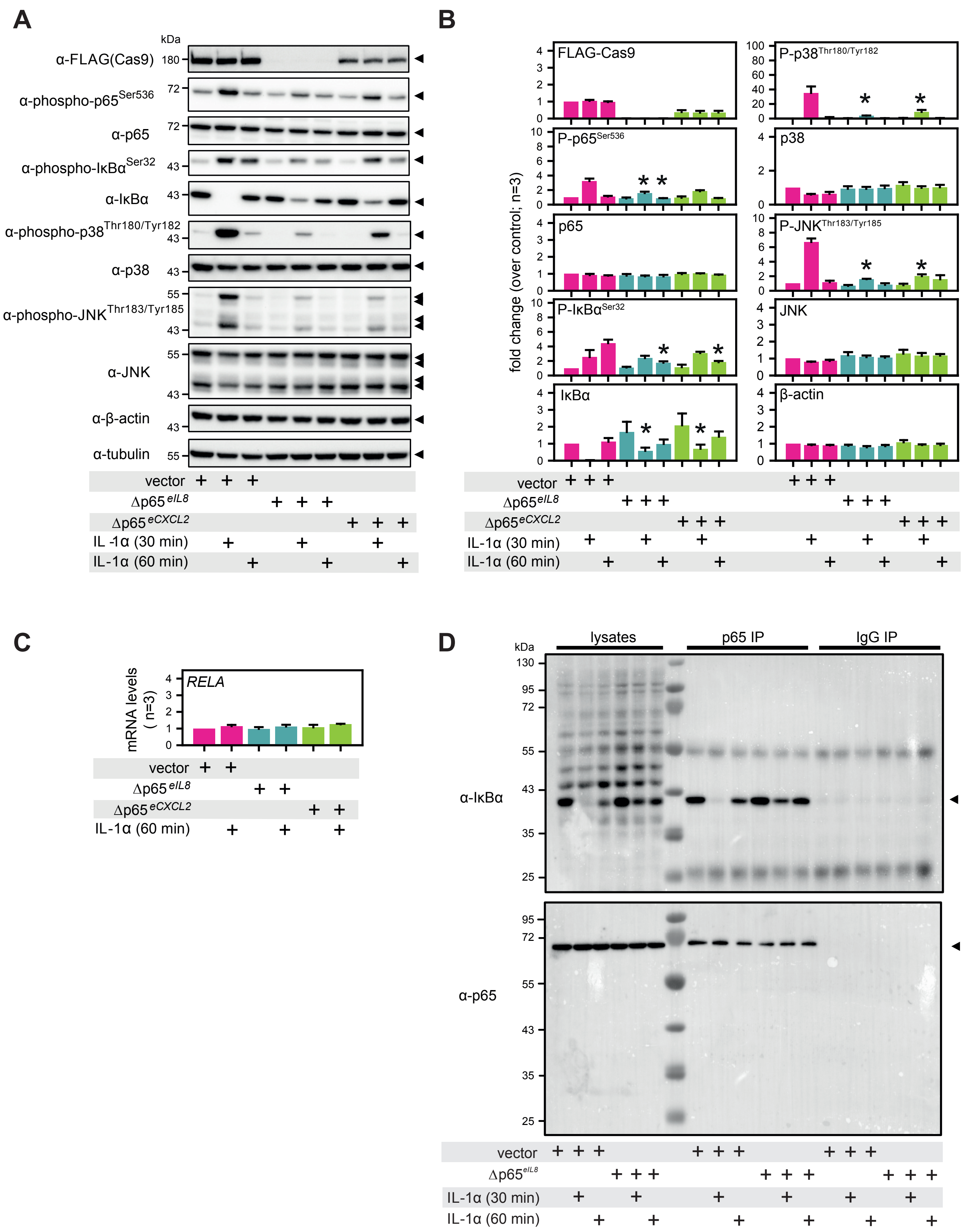

Appendix Fig S6

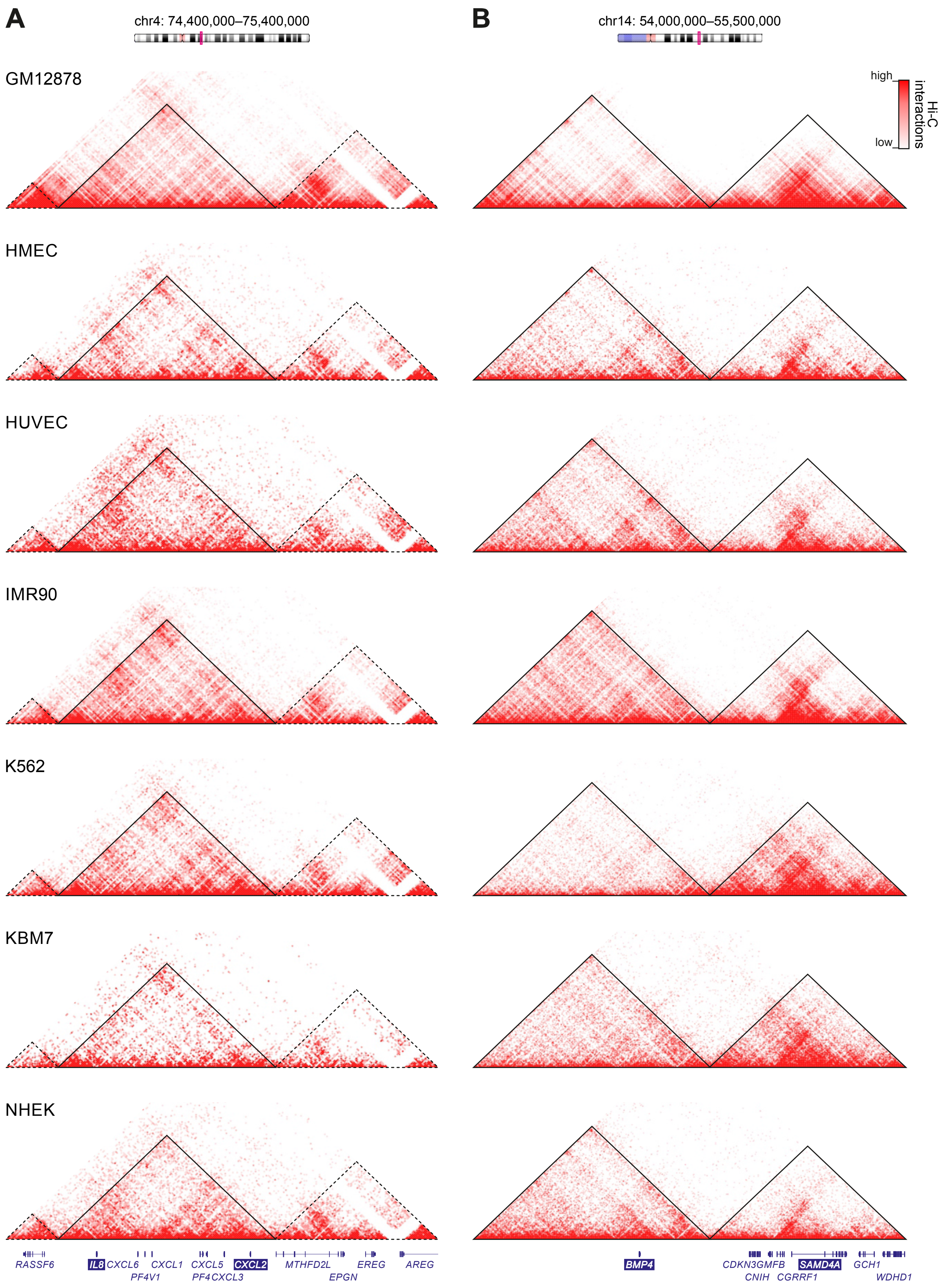

Appendix Fig S7

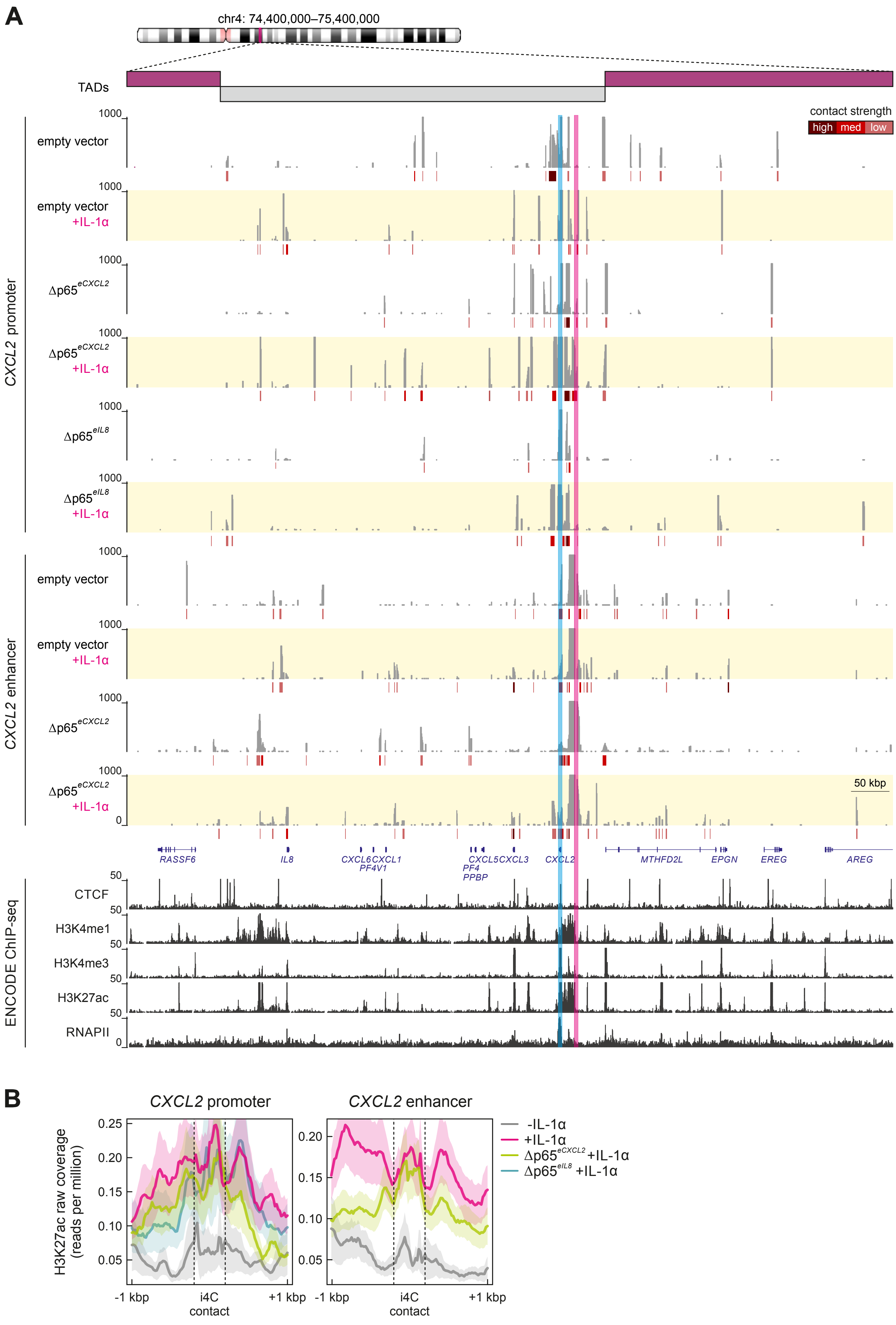

Appendix Fig S8

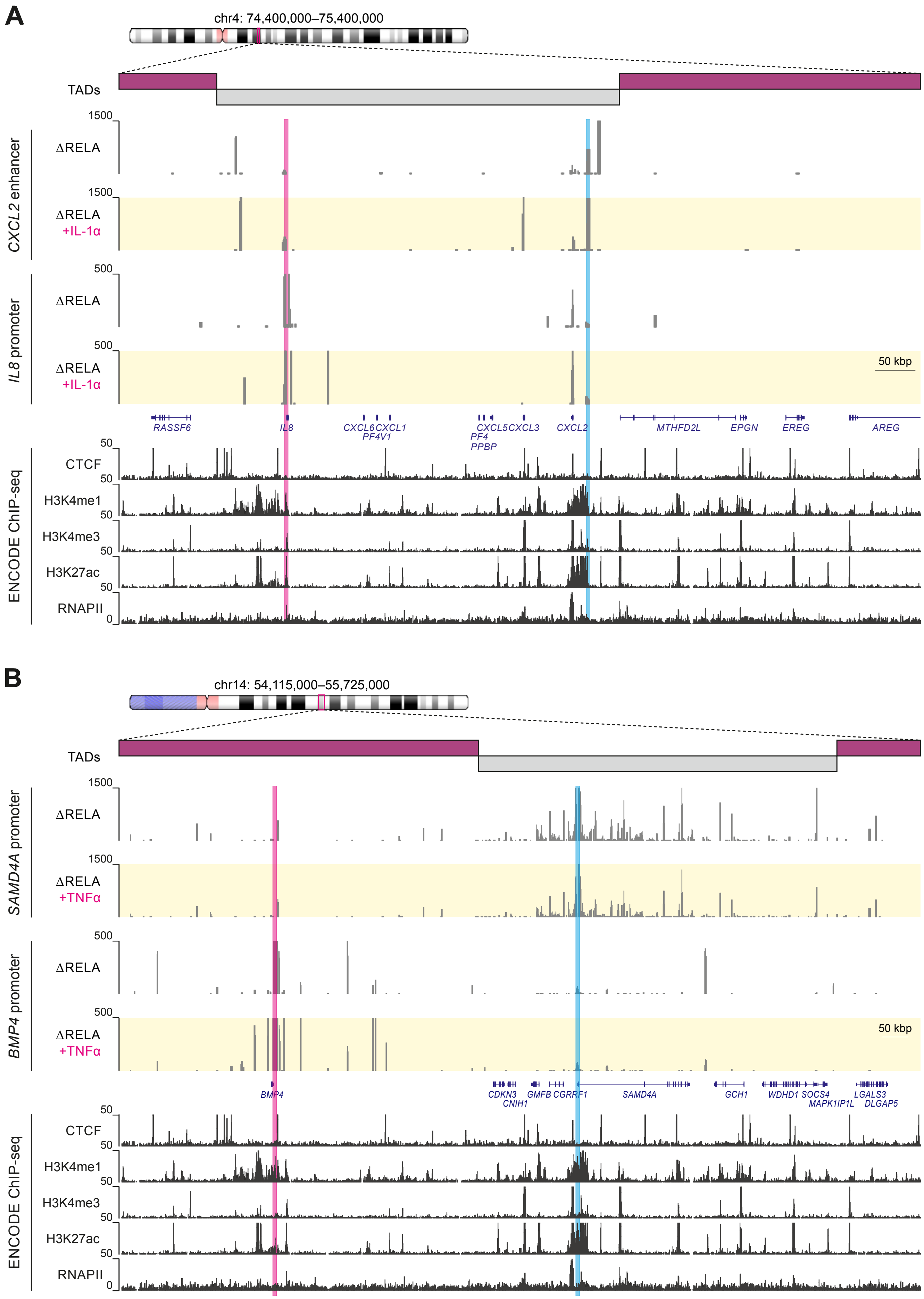

Supplement: Supplementary file 1 — Appendix [file EMBJ-39-e101533-s001.pdf]
