# Supplementary material for: Distinct IL‐1α‐responsive enhancers promote acute and coordinated changes in chromatin topology in a hierarchical manner
Source: EMBO J. 2019 Nov 7;39(1):e101533. doi: 10.15252/embj.2019101533 (PMC6939198; doi:10.15252/embj.2019101533)
Supplement: Supplementary file 2 — Expanded View Figures PDF [file EMBJ-39-e101533-s002.pdf]

Expanded View Figures

Figure EV1. Quantification of IL-1α-mediated enhancer modifications and p65 NF-κB binding in the human *IL8* and *CXCL2* chemokine loci.

Published ChIP-seq data from KB cells (Jurida *et al*, 2015; GSE64224 and GSE52470) were used to annotate active enhancers and p65 NF-κB recruitment in untreated cells compared to cells stimulated with IL-1α for 60 min ± TAKi. The browser views show ChIP-seq profiles for all conditions of the *IL8* and *CXCL2* chemokine loci. Gray areas highlight four chromatin regions with IL-1α-inducible H3K27 acetylation and p65 binding. As indicated by black horizontal bars, these chromatin regions were provisionally designated by us as "class II enhancers" (Jurida *et al*, 2015) to distinguish them from the entire repertoire of all active (i.e., H3K4me1- and H3K27ac-positive) enhancers. Vertical bars indicate predicted NF-κB motifs in the DNA sequence and the positions of all sgRNAs used in this study for CRISPRa (see Fig 6) or for enhancer or promoter deletions (see Figs 3 and EV4). Bar graphs show cumulative read counts across the four IL-1α-regulated and TAKi-sensitive class II enhancers. The two flanking enhancers with strongest p65 binding (2 and 4) are the ones investigated in detail in this study.

© 2019 The Authors

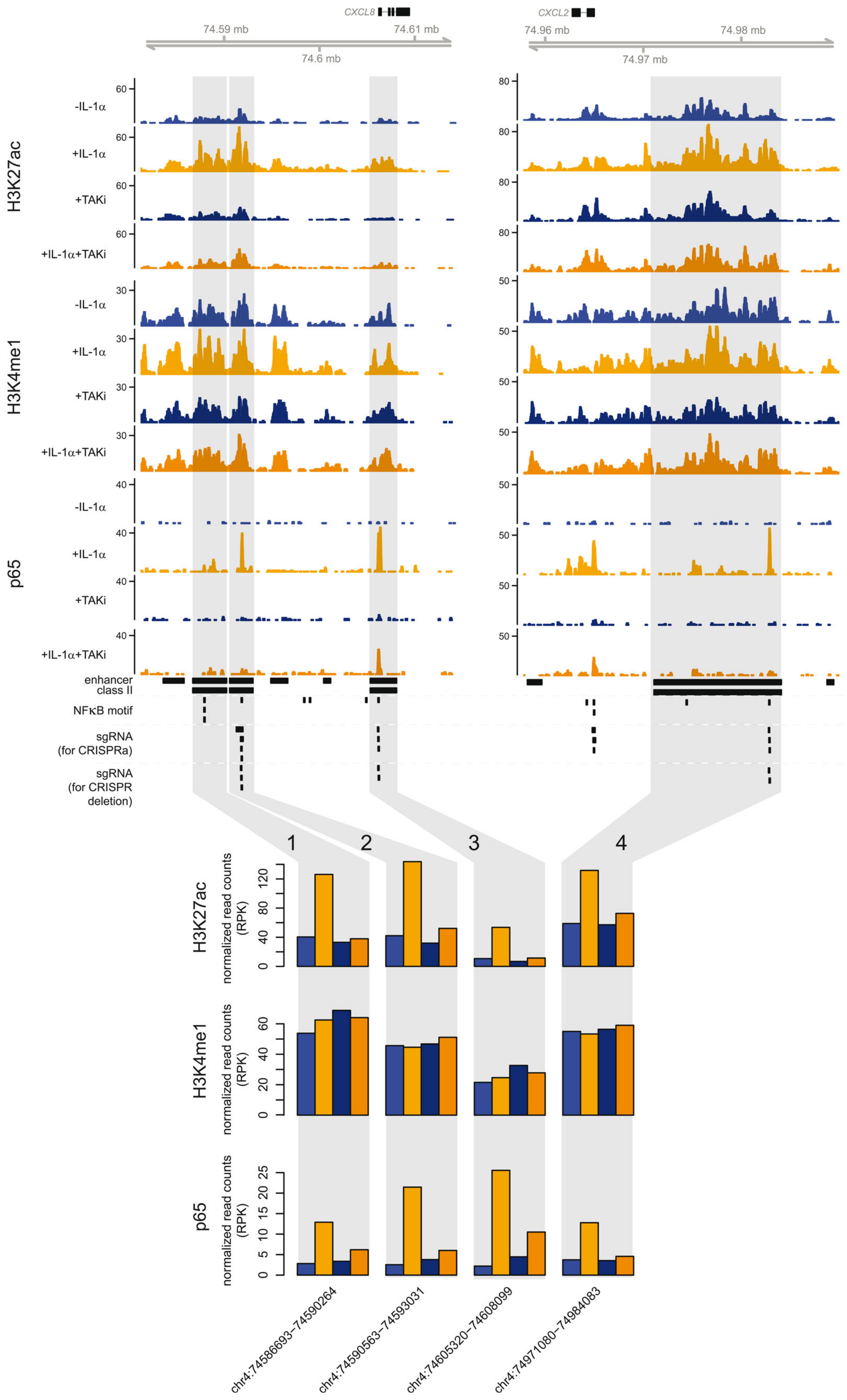

Figure EV1.

 © 2019 The Authors

Figure EV2. Analyses of mRNA stabilities and the global IL-1α gene expression response in enhancer-mutant HeLa.

A Empty vector controls or $\Delta p65^{eIL8}$, $\Delta p65^{eCXCL2}$, and $\Delta p65^{eIL8+eCXCL2}$ enhancer-mutant HeLa lines were stimulated with IL-1α for 60 min or were left untreated. Then, actinomycin D (5 μg/ml) was added to stop transcription and RNAs were immediately extracted (reference time point 0 min) or incubations were continued to monitor the decay of *IL8* and *CXCL2* mRNAs at different time points. RT–qPCR was performed from total RNA, and changes in mRNA expression were calculated and are depicted relative to the 0-min time point (mean levels ± SEM, normalized to *GUSB*; $n = 2$).

B Summary of microarray gene expression analysis performed in HeLa cells ± IL-1α stimulation for 60 min on control (empty vector; $n = 4$) and three p65 enhancer-deletion lines ($\Delta p65^{eIL8}/^{-CXCL2}/^{-IL8+CXCL2}$; $n = 2$). Differentially expressed genes were identified based on a moderated *t*-test (*P*-value < 0.05) and at least threefold change compared to the mean control levels (empty vector). All data are provided in Table EV1.

C Heatmap depicting fold changes ($\log_2$) in response to IL-1α for each sample over the mean of vector control levels (*blue to red*) for the top 93 IL-1α-regulated genes (> 3-fold, *P*-value < 0.05). Additionally, the effect of the three enhancer-mutant lines on the IL-1α response compared to the mean of IL-1α-stimulated vector controls is visualized (*purple to green*). Purple shades indicate suppression of IL-1α-activated genes.

D Gene ontology and KEGG terms associated with the 93 shared IL-1α-upregulated genes from panel (C) against the entire set of the 14,204 expressed genes.

© 2019 The Authors

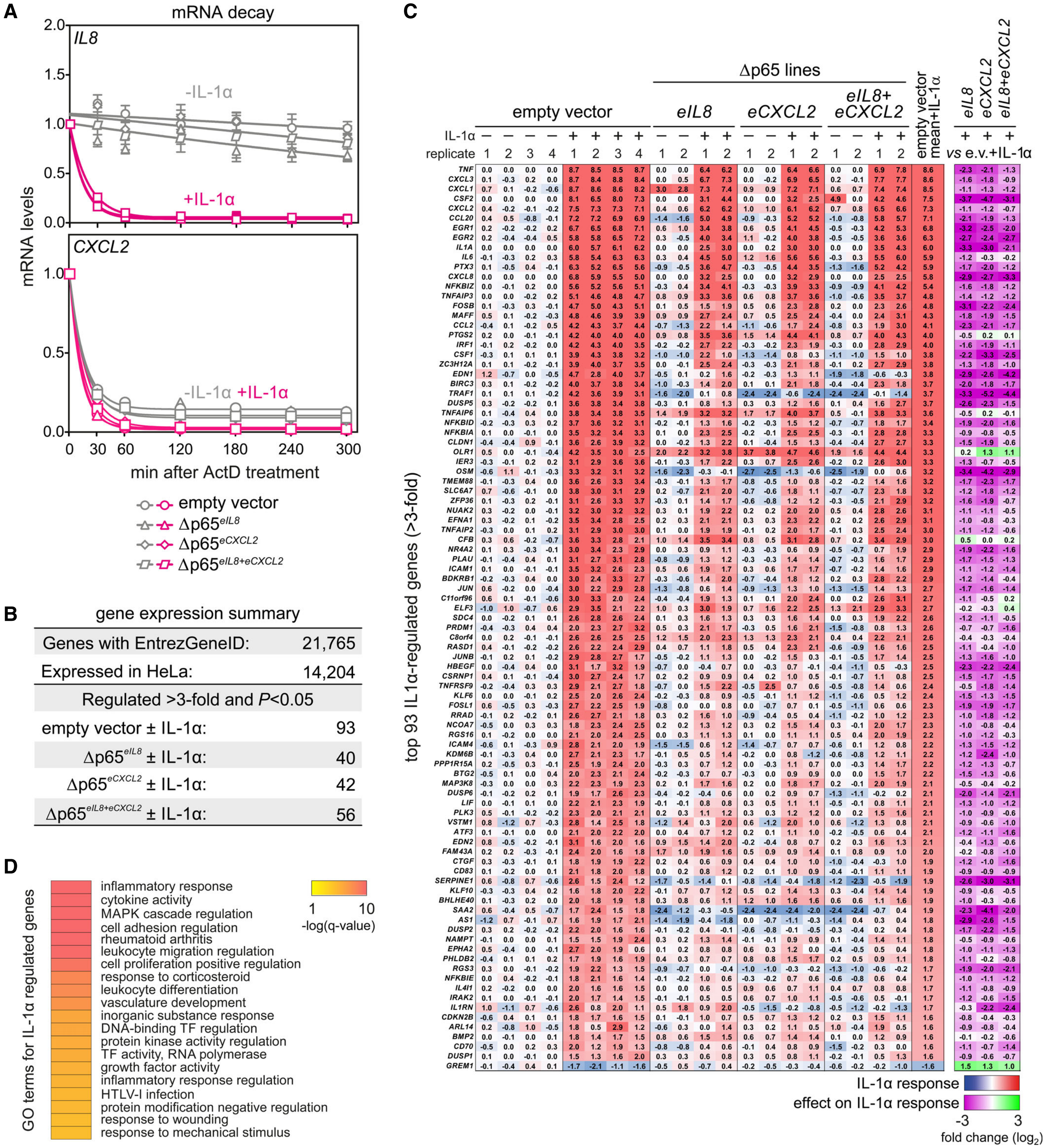

Figure EV2.

© 2019 The Authors

Figure EV3. Differential regulation of enhancers and promoters of IL-1α target loci.

A NF-κB (p65), histone marks, and RNA polymerase II enrichment at the *IL8* and *CXCL2* promoter and enhancer in control (empty vector) or mutated ($\Delta p65^{eIL8}$ and $\Delta p65^{eCXCL2}$) HeLa lines were assessed by ChIP-qPCR (mean enrichment over input ± SEM) at the indicated times after IL-1α stimulation. IgG, H3K27ac, H3K4me1, H3, and p65 ChIP-qPCR data are from three (vector, $\Delta p65^{eCXCL2}$) or four ($\Delta p65^{EIL8}$) independent experiments performed in duplicate; all others are from at least two independent experiments. *: significantly different to vector controls; $P < 0.01$, unpaired, two-tailed Student's *t*-test.

B As in panel (A), but for the promoters of the *IL6*, *CCL20*, *NFKBIA*, *CXCL1*, and *CXCL3* genes. Data are derived from at least two independent experiments (mean ± SEM, *$P < 0.01$, unpaired, two-tailed Student's *t*-test).

© 2019 The Authors

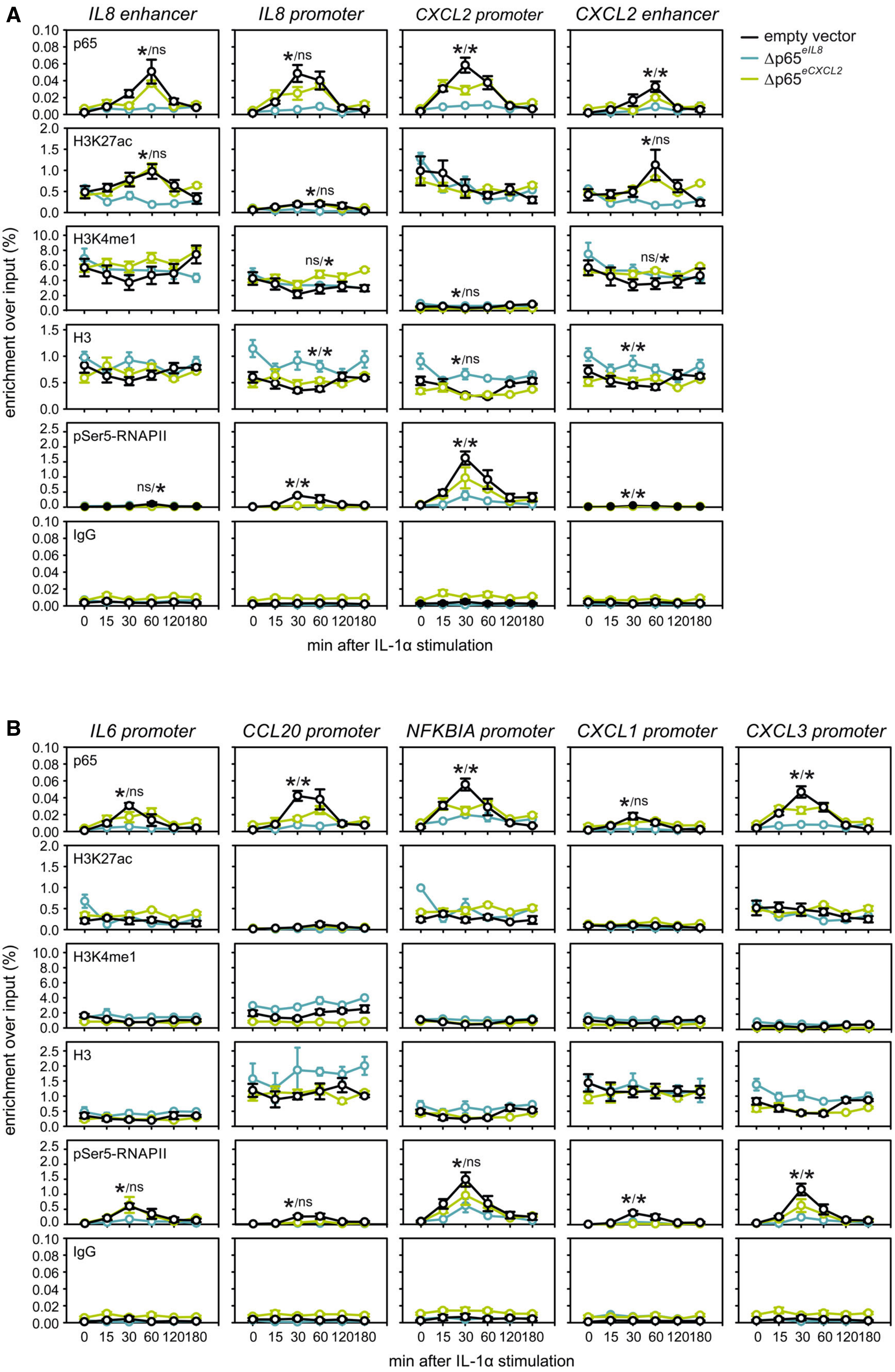

Figure EV3.

 © 2019 The Authors

Figure EV4. Deletion of the p65-binding site from the *IL8* promoter only affects *IL8* expression.

A Genome browser view of the *IL8* chemokine locus on human chromosome 4 showing H3K4me1, H3K4me3, H3K27ac, and RNA polymerase II ENCODE ChIP-seq profiles from HeLa-S3 cells relative to the *IL8* gene model (*blue*). The location of the deleted NF-κB binding site in the *IL8* promoter is indicated (*orange*), and Sanger sequencing of PCR amplicons generated from genomic DNA of the $\Delta p65^{pIL8}$ cell line shows removal of 57 bp.

B Immunoblot analysis of extracts from parental HeLa cells (wt), vector controls, or $\Delta p65^{pIL8}$ cells was compared to cells stably expressing a sgRNA targeting the *RELA* (Δ*RELA*) gene which encodes for the p65 NF-κB subunit in the presence (+) or absence (−) of IL-1α stimulation for 30 min or 60 min. Antibodies against p65 confirm the strong suppression of p65 in Δ*RELA* cells. Antibodies against P(S536)-p65, P-IκBα, and IκBα reveal normal IL-1α-mediated signaling in $\Delta p65^{pIL8}$ cells compared to control cells, while Δ*RELA* cells show reduced levels of the IL-1α and the p65 target gene IκBα. β-Actin levels provide a loading control. Cas9 antibodies reveal the levels of FLAG-Cas9 present in the stable cell cultures.

C mRNA levels of seven IL-1α-responsive genes in parental (wt), control (empty vector), or promoter-mutant ($\Delta p65^{pIL8}$) HeLa lines were assessed by RT–qPCR (mean levels ± SEM, n = 4) at 60 min after IL-1α stimulation. Asterisks show significance of changes in basal and IL-1α-stimulated conditions compared to the corresponding vector control samples ($P < 0.01$, Mann–Whitney rank-sum test).

D The same mRNAs as in (C) were determined in Δ*RELA* cells from four independent experiments (mean levels ± SEM).

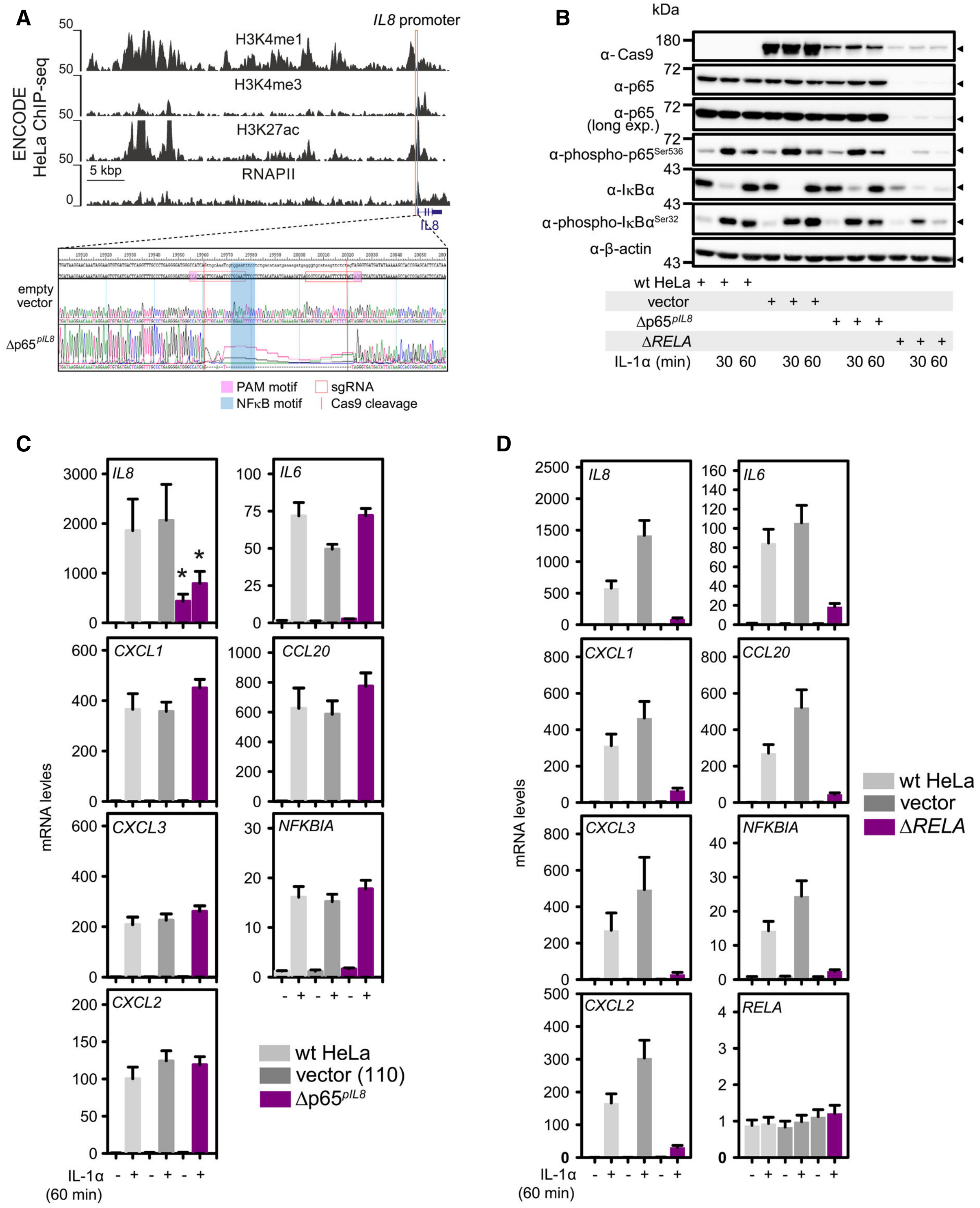

Figure EV4.

 © 2019 The Authors

Figure EV5. Complex enhancer hierarchies in primary human endothelial cells.

A i4C profiles in the 1.6 Mbp around the *BMP4* and *SAMD4A* loci on chromosome 14 (*ideogram*) ± TNFα stimulation for 60 min. Data were generated using the *SAMD4A* (*blue highlight*) and *BMP4* promoters (gray *highlight*) or enhancers (*dotted rectangles*) as viewpoints, and profiles are shown aligned to gene models (*blue*) and to ENCODE ChIP-seq profiles from primary HUVECs. The breadth of topologically associating domains (TADs) in the locus is indicated above (*rectangles*). ΔeBMP4 and ΔeSAMD4A indicate i4C data generated using primers specifically targeting the respective enhancer deletions in the population.

B *Left*: electrophoresis profiles of wild-type (wt) and truncated amplicons (*scissors*) from CRISPR-edited HUVEC populations, where the upstream *BMP4* enhancer was deleted in > 30% alleles. *Right*: relative changes in nascent (intronic) RNA levels of the *BMP4* (*gray*), *SAMD4A* (*blue*), and *CXCL2* TNFα-inducible genes (*black*) between wild-type and ΔeBMP4 cell populations in the presence or absence of TNFα stimulation. Shown are mean values ± SD. *P-value < 0.05; unpaired, two-tailed Student's t-test ($n = 2$).

C As in panel (B), but for HUVECs carrying ~12% alleles lacking the *SAMD4A* intronic enhancer. Shown are mean values ± SD. *$P < 0.05$; unpaired, two-tailed Student's t-test ($n = 2$).

D Bar plot showing the distribution of split reads that mapped to each chromosome from multi contact (MC)-i4C experiments using the *SAMD4A* promoter (*blue*), *SAMD4A* enhancer (*white*), or *BMP4* enhancer (*gray*) as a viewpoint; unmapped reads are also shown.

E Bar plot showing the number of interacting fragments contained in each analyzed MC-i4C read. *Inset*: counts for the number of useful reads and fragments per each viewpoint.

F MC-i4C profiles in the 2 Mbp around the *BMP4* and *SAMD4A* loci on chromosome 14 (*ideogram*) ± TNFα stimulation for 60 min. Data were generated using the *SAMD4A* promoter (*blue highlight*) and enhancer (*dotted rectangle*) or the *BMP4* promoter (*gray highlight*) as viewpoints, and profiles are shown aligned to gene models (*blue*) and to ENCODE ChIP-seq profiles from primary HUVECs.

Source data are available online for this figure.

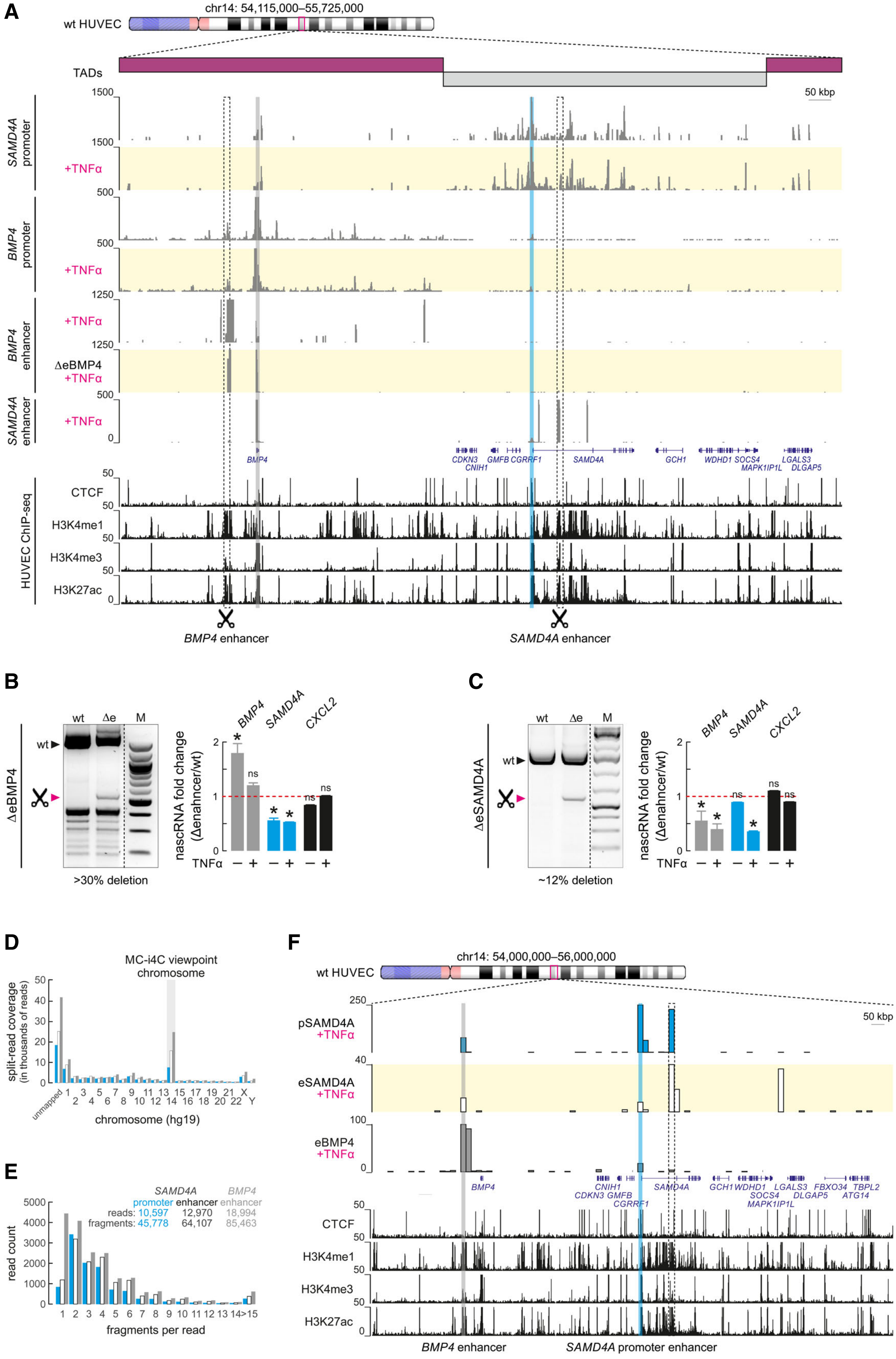

Figure EV5.

 © 2019 The Authors
